# Assessment of dynamic cerebral autoregulation in near-infrared spectroscopy using short channels: A feasibility study in acute ischemic stroke patients

**DOI:** 10.3389/fneur.2022.1028864

**Published:** 2022-11-21

**Authors:** Sabeth Becker, Franziska Klein, Katja König, Christian Mathys, Thomas Liman, Karsten Witt

**Affiliations:** ^1^Department of Neurology, School of Medicine and Health Sciences, Carl von Ossietzky University of Oldenburg, Oldenburg, Germany; ^2^Neurocognition and Functional Neurorehabilitation Group, Neuropsychology Lab, Department of Psychology, Faculty of Medicine and Health Sciences, University of Oldenburg, Oldenburg, Germany; ^3^University Clinic for Neurology, Evangelical Hospital, Oldenburg, Germany; ^4^Institute of Radiology and Neuroradiology, Evangelical Hospital, Oldenburg, Germany; ^5^Research Centre Neurosensory Science, Department of Human Medicine, Faculty of Medicine and Health Sciences, University of Oldenburg, Oldenburg, Germany

**Keywords:** acute ischaemic stroke (AIS), cerebral autoregulation (CA), cerebral blood flow (CBF), head of bed positioning, low-frequency oscillation (LFO), near-infrared spectroscopy (NIRS), very low-frequency oscillations, short channel

## Abstract

**Introduction:**

In acute ischemic stroke, progressive impairment of cerebral autoregulation (CA) is frequent and associated with unfavorable outcomes. Easy assessment of cerebral blood flow and CA in stroke units bedside tools like near-infrared spectroscopy (NIRS) might improve early detection of CA deterioration. This study aimed to assess dynamic CA with multichannel CW-NIRS in acute ischemic stroke (AIS) patients compared to agematched healthy controls.

**Methods:**

CA reaction was amplified by changes in head of bed position. Long- and short channels were used to monitor systemic artery pressure- and intracranial oscillations simultaneously. Gain and phase shift in spontaneous low- and very low-frequency oscillations (LFO, VLFO) of blood pressure were assessed.

**Results:**

A total of 54 participants, 27 with AIS and 27 age-matched controls were included. Gain was significantly lower in the AIS group in the LFO range (i) when the upper body was steadily elevated to 30. and (ii) after its abrupt elevation to 30°. No other differences were found between groups.

**Discussion:**

This study demonstrates the feasibility of NIRS short channels to measure CA in AIS patients in one single instrument. A lower gain in AIS might indicate decreased CA activity in this pilot study, but further studies investigating the role of NIRS short channels in AIS are needed.

## Introduction

Cerebral blood flow is substantially regulated by the mechanism of cerebral autoregulation (CA). CA continuously adjusts vascular resistance and diameter to intercept systemic blood pressure (BP) changes. For that, it uses myogenic mechanisms (1–4), the autonomic nervous system (2–7), and CO2 partial pressure changes (8). CA protects brain tissue from reduced perfusion and hence brain ischemia and remains unaltered with aging and hypertension (9, 10). However, major impairment of CA is frequent in AIS (11–13). The effect of postural changes on cerebral blood flow (14–16), e.g., seen in controlled changes in an orthostatic maneuver [changes in upper body position, head of bed (HOB)] serves as an indicator for severity of CA impairment. This orthostatic maneuver could be analyzed with spontaneous blood pressure oscillations that provide comprehensive information on CA (17).

Near-infrared spectroscopy (NIRS) measures hemodynamic changes. By using optodes (i.e., light sources and light detectors) near-infrared light is transmitted through the head surface into the tissue. Hence, hemoglobin absorption can be quantified as concentration changes in oxygenated and deoxygenated hemoglobin *in vivo* (18). NIRS provides information on both scalp perfusion, which represents regulation of the extracerebral vessels, and cerebral perfusion, which is constantly modified by CA. Hence, NIRS is an excellent tool to assess the differences between extra- and intracerebral vascular autoregulation. It is a useful, non-invasive and investigator-independent bedside measurement for CA (19). Global blood flow regulation processes are the main source of physiological signals measured superficially by NIRS in the scalp (20, 21), and is often considered as disturbing factor. In the past years, extracerebral NIRS signals have been eliminated using short distance correction as a filter to obtain only the adjusted intracerebral signals (22–24).

NIRS records cerebral changes in the local oxygenated and deoxygenated hemoglobin concentrations on the cerebral cortex. In this signal, systemic arterial BP oscillations can indirectly be displayed (25–27), and subdivided into frequency ranges. Each range provides information about different aspects of CA: Low-frequency oscillations (LFO or Mayer waves, ~0.1 Hz) reflect the local myogenic activity in the terminal arterioles and are further influenced by sympathetic tone and control mechanisms (28, 29). Very low-frequency oscillations (VLFO or B-Waves) range in the minute range (30). Upper VLFO (~0.05 Hz) stem from large arterioles under neurogenic innervation (31–33) and are modulated by arterial CO2 partial pressure (34, 35). Lower VLFO (~0.01 Hz) are endothelial dependent and thus connected to metabolic changes in the microvessels (36). Intact CA constantly modifies spontaneous dynamic oscillations resulting in higher desynchronization of intra- and extracranial BP oscillations. However, an impaired CA (as in AIS) is unable to modulate BP, and therefore intracranial oscillations passively and synchronously follow those of systemic BP (37).

So far, in NIRS studies on (dynamic) CA, the use of an external tool that measures systematic blood pressure variations were necessary: While intracranial BP changes have been assessed by NIRS or transcranial doppler, systemic BP changes were recorded using a finger plethysmograph (26, 38–41) or an arterial or aortic catheter (32, 42, 43). Afterwards, these independent datasets needed to be synchronized to detect modulation by CA. However, this synchronization, accurate to milliseconds, is complex and error-prone. Therefore, one main aim of the present study was to address this using the short channel signal instead.

To assess the synchronicity of extra- and intracerebral oscillations and, therefore, the extent of modification by CA, fourier analyses such as discrete fourier transform (DFT) are commonly used (17). By DFT, measuring points are periodically continued into sinus curves. Input-sinusoids (=systemic BP-signal) are transformed into sinusoids of the same frequency at the output (=intracranial BP-signal) but with a different amplitude (*gain*) and shifted in time (the *phase shift* of the response). In this study, the synchronicity of two signals will be assessed: The oscillation in the short channels (input signal), which detect the systemic oscillations of the ABP *via* the scalp, and the oscillation modified by CA, which is measured *via* long channels (output signal).

The feasibility of NIRS has rarely been tested in AIS patients. The aims of this study are (i) to detect CA changes in AIS patients in comparison to matched-healthy controls and (ii) to test if these changes can be assessed using a single NIRS system, including short- and long channels, in an acute stroke unit setting.

## Materials and methods

This study reports a prospective analysis of CA measurements in AIS patients and a healthy control group with multichannel CW-NIRS. In this study, scalp perfusion is monitored during upper-body position changes with extracerebral short channels to (i) correct the signals generated from long channels and (ii) record systemic BP-oscillations. It was hypothesized that long channels could be used to assess intracranial blood pressure signals and short channels to simultaneously assess systemic BP signals.

### Study population

**Figure 1:**
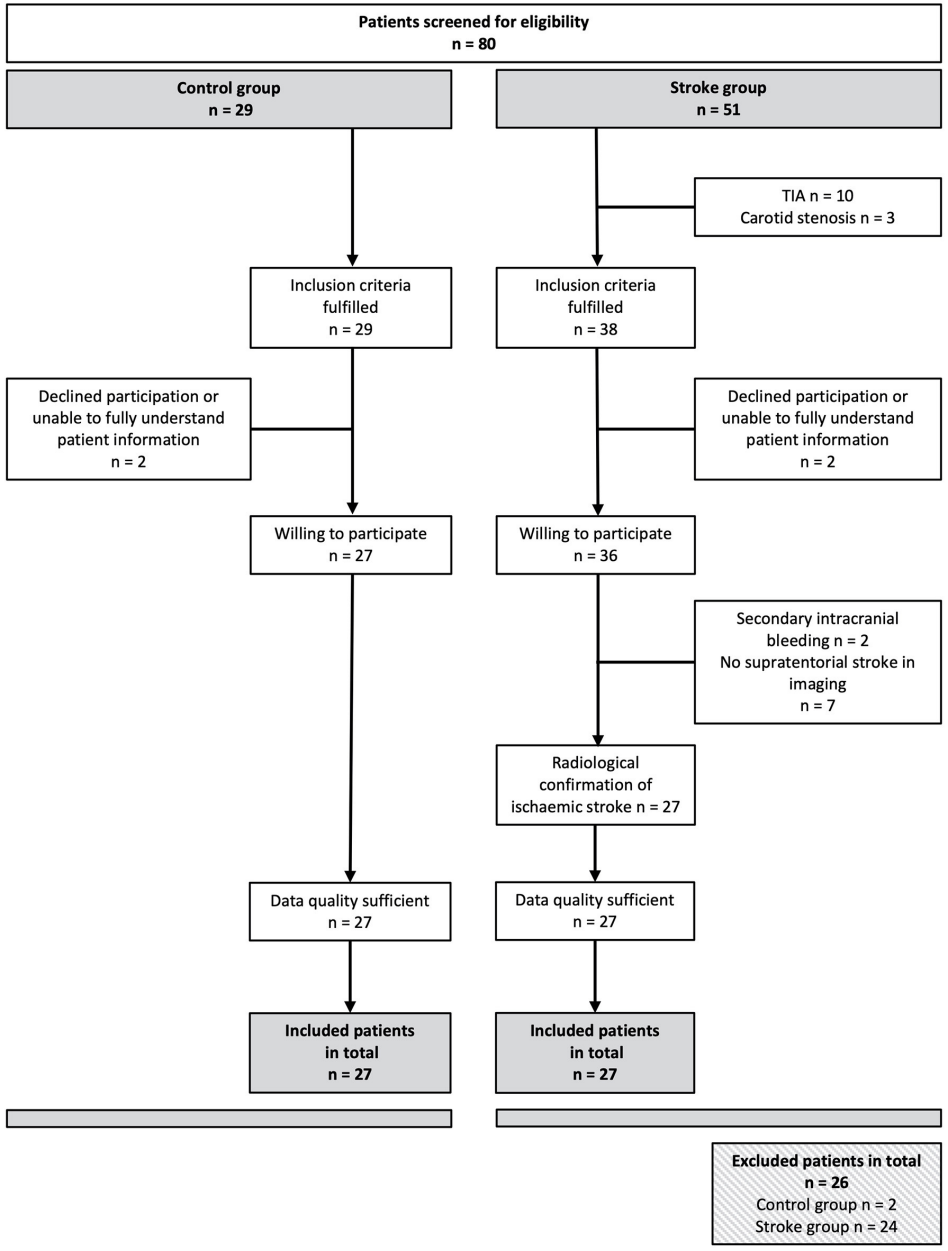
Flow chart of the recruitment of subjects. Of 80 patients initially screened, 54 could be included in the study. Both the control group and the stroke group included 27 people each.

Patients with AIS were consecutively recruited from the stroke unit at the evangelical hospital at the university of Oldenburg in 2020. Inclusion criteria were age >18 years, acute stroke according to the WHO criteria restricted to symptoms in the territory of the middle cerebral artery (MCA), and evidence of MCA stroke lesion detected in MRI or CT, symptom onset within 48 h. Exclusion criteria were: known autonomic dysfunction, subdural hematoma or subarachnoid hemorrhage, ICU stay >48 h, and/or carotid stenosis >40% (44). Eighty AIS were screened for eligibility. Twenty-seven patients with AIS were included in the study. For patient inclusion flow, see Figure 1. Healthy controls were recruited from volunteers from the university and matched with patients by age (±3 years). For controls, exclusion criteria were a history of any neurological or vascular disease. All participants gave their written informed consent. The study was approved by the medical ethics committee of the University of Oldenburg. It was conducted in accordance with the Declaration of Helsinki. The study has been registered at DRKS, the German WHO primary registry for all clinical trials conducted in Germany (ID: DRKS00029613).

### Data acquisition

#### Near-infrared spectroscopy recordings

**Figure 2:**
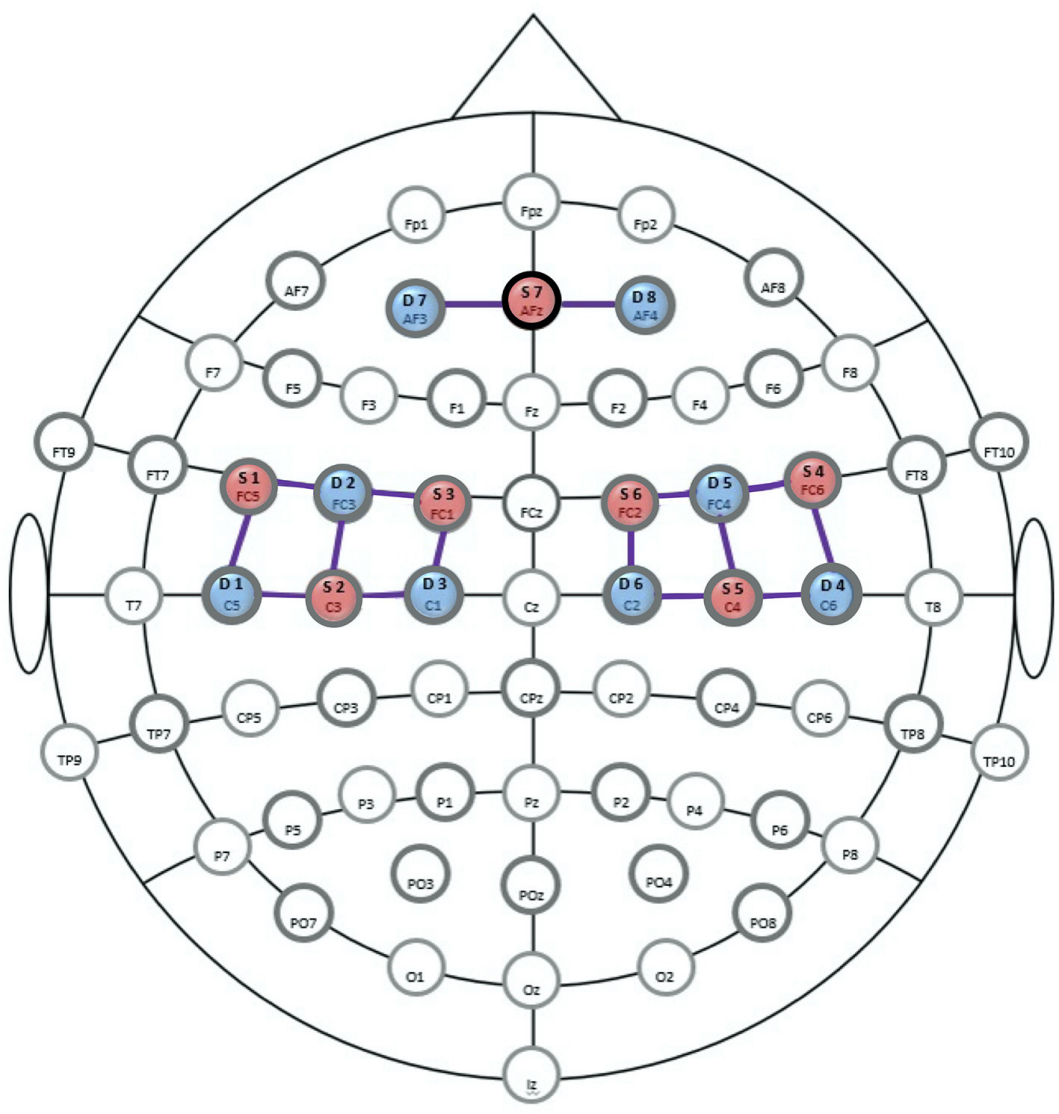
Optode-setup for the near-infrared spectroscope (NIRS). Positions of the sources are shown in red (S1-7), those of the detectors in blue (D1-8). Long channels (LDC) are marked as connecting lines. Positions of the extended 10/20 EEG system are shown as landmarks for orientation. F, frontal; T, temporal; O, occipital; C, central; Iz, inion (central). Source: Figure generated from a NIRx-template (45).

NIRS signal was acquired noninvasively using optodes, that is, light sources and detectors. Records were made with CW-NIRS device NIRx NIRScout 816 and its corresponding recording software NIRStar (NIRx Medizintechnik GmbH, Berlin, Germany), which operates at wavelengths of 760 and 850 nm. The sampling rate was 7.8125 Hz. Seven sources and nine detectors for long channels were used, as well as seven short channel detectors attached to the sources. The distance between sources and long channel detectors was ~3.0 cm. Distance between a source and its short channel detector was 0.8 cm. A symmetrical bilateral optode setup was chosen over the mid cerebral artery supply area (ACM). In addition, data were collected *via* two frontal channels in the supply area of the anterior cerebral artery (ACA). Individualization and displacement of the measurement points for each subject was not performed to ensure that the monitoring device could be attached quickly in accordance with the daily clinical routine and to ensure a high degree of comparability between the groups. Measurement points of the NIRS optodes were located bihemispherically on C1–C6, FC1–FC6, F3, F4, and Fz, resulting in seven source and eight detector channels (see Figure 2).

#### Experimental protocol

**Figure 3:**
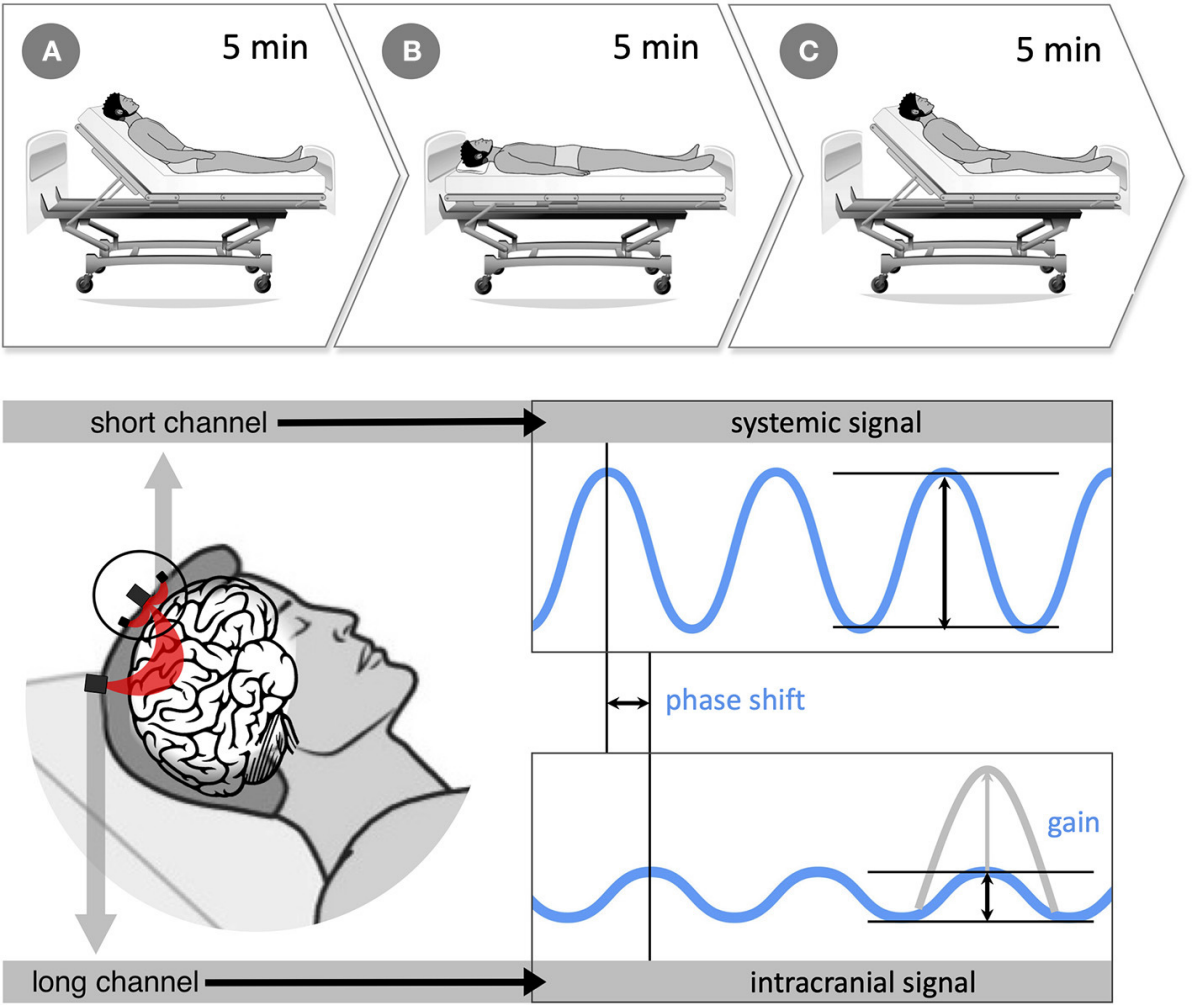
**(Top)** experimental protocol with 3-, 5-min recording periods (sections A 30°, B 0°, and C 30°). First, the baseline was recorded for 5 min with the upper body upright at 30° (section A), then the upper body was passively lowered to 0° in the horizontal position (section B). Finally, after another 5 min, the upper body was passively raised to 30° (section C). The entire recording lasted 15 min. **(Bottom)** data acquisition and processing: near-infrared spectroscopy (NIRS) measures hemodynamic changes with optodes transmitting near-infrared light through the head surface into the brain tissue. Long-distance channels have ~3.0 cm between the light source and its detector and provide information on intracerebral hemodynamics. Short-distance channels are located directly next to each light source (0.8 cm) and record systemic blood pressure (BP) changes in the scalp. Systemic and intracranial signals were set in relation using discrete fourier transform to obtain information on cerebral autoregulation (CA). Like that, systemic and intracranial BP curves can be compared in amplitude (*gain*) and shift in time (*phase shift*). The more asynchronous the two signals, the more CA modulates the BP signal. Source: Own figure.

Before recording, vital parameters, oxygenation, and a physical examination were taken. The patients were passively positioned in an electronically adjustable bed on stroke unit during measurement. Data were recorded for 15 min. This measurement period, divided into sections of 5 min each, corresponds to the widely used standard used in comparable head-of-bed studies on blood pressure oscillation (14, 15, 41, 46–49). It represents a trade-off between the amount of data acquired and the stress on the patients. For 5 min each, data were collected with the head-of-bed (HOB) raised to 30° (section A, baseline measurement), in a flat position lowered to 0° (section B), and again in 30° elevated position (section C). Sessions were recorded in one dataset per person for all HOB-maneuvers to avoid inaccuracies in the time axis. The correct angles were determined using a protractor. An overview of the experimental protocol is given in Figure 3. Measurements were taken within the first 24 h after stroke onset.

### Data processing

Preprocessing was performed in MATLAB (MathWorks, version R2018b 9.5.0.944) (50) using the NIRS Brain AnalyzIR toolbox48 and custom-made scripts (51). Quality was checked visually and electronically. For this purpose, a gain setting of 8 and a coefficient of variation of a maximum of 15% was accepted. Only data of those participants were further processed if at least four long- and short channels remained for analysis. No participant had to be excluded due to poor data quality. Raw data were transformed into optical density changes and finally into hemoglobin concentration changes. Then, movement artifacts were corrected using the movement artifact reduction algorithm (MARA) (52). Motion artifact segments were automatically identified on a channel-by-channel basis [using the function hmrMotionArtifactByChannel from the Homer2 NIRS Processing package with a threshold in changes of the amplitude of 0.4, a threshold in changes of the standard deviation of 50, a time period of 1, and a tMask of 1 (53)]. This provided a compromise between the number of motion artifacts identified in noisier data series and the number identified in less noisy data series. Next, the optical density changes were bandpass-filtered with a third-order Butterworth filter at a cutoff of 0.15 Hz and 0.005 to eliminate heart rate, respiratory rate, as well as background noise, baseline drifts, and artifacts due to optode dislocation. Data were then converted into hemoglobin concentration changes using the modified Beer-Lambert law [partial pathlength factor (=0.1) = differential pathlength factor (=6) ^*^ partial volume factor (=1/60)]. All long channels were corrected for systemic activity contributions by applying the short separation regression by Saager and Berger (54). Here, for each long channel, the spatially closest short channel was used for correction. Finally, signals were normalized to z-scores by subtraction of the mean and division by the standard deviation of the baseline. All further analyses were based on oxygenated hemoglobin (oxyHb) only.

The signal components of the short channels are noise in the signal of the long channels (55). Therefore, these components were removed from the long channel signal to minimize noise. In this study, short channel signals were used in two ways: As a correction to subtract noise from the long channels and on their own in filtered form as a representative of the systemic oscillation and its changes during an orthostatic maneuver. The channels were averaged over the respective hemisphere (left or right) and subdivided into the measurement sections A 30°, B 0°, and C 30°. The first and last 30 s after position change were excluded from the analysis to avoid signal contamination during position change. The degree of synchronicity between systemic and cerebral arterial BP oscillations was determined using DFT. DFT was used to decompose the NIRS signal into the different frequency ranges and to relate the systemic BP signals to the intracerebral BP signals (see introduction). For the low-frequency oscillations (LFO), a range of 0.07–0.15 Hz was used, 0.02–0.07 Hz for upper VLFO, and 0.0095–0.02 Hz for lower VLFO. Analyses were performed separately for each frequency band. Gain and phase shift were calculated with DFT as parameters of synchronicity (17, 56). The oxyHb gain was defined as the gain of the extracranial short channel-signal compared to the intracranial long channel signal. The closer the result is to 1, the more similar the two signals are. OxyHb phase shift was defined as the phase shift between the extracranial short channel-signal and intracranial long channel signal. The time delay between the systemic and cerebral oscillations is given as an angle in degrees. If both signals have their zero crossings simultaneously, the angle is 0°. A negative value means that input oscillation occurs earlier than output oscillation.

### Statistical analysis

Statistical analyses were performed with SPSS Statistics (IBM Corp. 2020. IBM SPSS Statistics for Macintosh, v 27.0. Armonk, NY: IBM Corp). Continuous data from the two groups were compared using the student's test or Mann-Whitney *U*-test depending on normality of distribution. Categorical variables were compared using the Pearson chi-squared test. For the stroke group, data from the impaired hemisphere were analyzed. Matched controls were analyzed corresponding to their stroke-match patient (e.g., AIS in the right hemisphere in an AIS patient means that the right hemisphere of the matched healthy control is analyzed). Furthermore, the unaffected hemispheres of the stroke group were tested against a matched healthy hemisphere of the control group. This way, a differentiation was possible of whether CA is primarily impaired in the affected hemisphere or in the healthy hemisphere as well. For this purpose, an analysis was carried out with a 2 × 3 mixed analysis of variance (ANOVA) with a between-subjects factor *group*_*HealthyHem*_ (healthy hemisphere stroke, control group) and a within-subjects factor *upper body position* (A 30°, B 0°, and C 30°).

Furthermore, a distinction was made between strokes located directly below the optodes and those outside the direct measurement area. The aim was to differentiate whether a significant result is only determined by those patients with ischemia directly in the measurement area. For this purpose, for both gain and phase shift as dependent variables, a 2 × 3 repeated-measures ANOVAs with one within-subjects factor *area* (stroke directly under measurement area, stroke outside measurement area) and one within-subjects factor *upper body position* (A 30°, B 0°, and C 30°) was performed.

For statistical analyses, mixed ANOVAs and repeated-measures ANOVAs were conducted, and Bonferroni correction was applied to adjust for multiple comparisons. To investigate a difference in CA modulation between the groups during changes in upper body position, three 2 × 3 mixed ANOVAs with between-subjects factor *group*_*AISHem*_ (stroke, controls) and within-subjects factor *upper body position* (A 30°, B 0°, and C 30°) were applied for both gain and phase shift as the dependent variable. These mixed ANOVAs were performed each for a separate frequency band: (1) LFO, 0.07–0.15 Hz, (2) upper VLF, 0.02–0.07 Hz, and (3) lower VLFO, 0.0095–0.02 Hz. This was done to distinguish between the different mechanisms of action of CA.

## Results

### Population characteristics

Twenty-seven patients and twenty-seven healthy controls were included in the final analysis. The participants' characteristics are presented in Table 1. The mean cross-gender age of the total cohort was 69.4 years (SD ± 13.1 years). There was no significant difference between the groups in terms of age, gender, handedness, previous illnesses, and smoking behavior. No significant differences were found for antiplatelet therapy, statins, and antidiabetics, whereas frequency of patients' antihypertensive medication was significantly higher in the stroke group (*p* = 0.02). In the stroke group, ischemic tissue was located directly below the NIRS measurement optodes in 40.7% of the cases. In 59.3%, the AIS was located outside NIRS optodes. See Table 1 for demographics and details of the patient population.

**Table 1:** Patient characteristics.

**Patient characteristics**	**Stroke**	**Control**	**Comparison**
	**(*n* = 27)**	**(*n* = 27)**	**(Sig.) *P***
	**Mean (±SD)**	**Mean (±SD)**	
Mean age in years—no. (SD)	73,30 (± 10,55)	65,50 (± 10,55)	>0.05
Handedness—no. right (%)	26 (96.3)	25 (92.6)	>0.05
Sex—no. female (%)	14 (51.9)	16 (59.2)	>0.05
Diagnoses^(a)^
Atrial fibrillation—no. (%)	6 (22.2)	2 (7.4)	>0.05
ACI-stenosis^(b)^–no. (%)	4 (14.8)	1 (3.7)	>0.05
Arterial hypertension—no. (%)	13 (48.1)	10 (37)	>0.05
Diabetes mellitus—no. (%)	8 (29.6)	3 (11.1)	>0.05
Smoking^(c)^–no. (%)	6 (22.2)	4 (14.8)	>0.05
Medication
Aspirin—no. (%)	7 (25.9)	3 (11.1)	>0.05
Statins—no. (%)	7 (25.9)	3 (11.1)	>0.05
Antidiabetics^(d)^–no. (%)	6 (22.2)	3 (11.1)	>0.05
Antihypertensives—no. (%)	20 (74)	9 (11.1)	0.020
Etiology (TOAST classification)—no. (%)
Large artery atherosclerosis—no. (%)	2 (7.4)		
Cardioembolism—no. (%)	17 (63.0)		
Small artery occlusion—no. (%)	6 (22.2)		
Other etiology—no. (%)	0 (0)		
Undetermined etiology—no. (%)	2 (7.4)		
Hemisphere—no. right (%)	12 (44.4)		
Thrombolytic therapy (rtPA)—no. (%)	6 (22.2)		
Mechanical thrombectomy—no. (%)	5 (18.5)		
Median NIHSS^(e)^—no. (%)	6		
Vital signs^(e)^
SpO2 (%)	98		
Heart rate (bpm)	74		
NIBP (mmHg)	134 / 73		

Sig., significance; ACI, internal carotid artery; NIHSS scale, National Institutes of Health Stroke Scale; TOAST, Trial of Org 10172; NASCET, North American Symptomatic Carotid Endarterectomy Trial; (a) pre-existing conditions at the time of recording. (b) Presence of stenosis of the internal carotid artery of maximum 40% occlusion according to NASCET definition. (c) Current nicotine abuse or abuse at the time of incidence. (d) Insulins and/or oral antidiabetics. (e) At the time of recording. Group comparison was made using T-test (age) and chi-square tests. Data are presented as counts in absolute numbers and percentage if not stated otherwise.

### Experimental results

To exclude selection bias, a comparison of the unaffected hemisphere in AIS patients with the control group was performed with a 2 × 3 mixed ANOVA for *area* (stroke directly under measurement area, stroke outside measurement area) x *upper body position* (A 30°, B 0°, and C 30°). Here, no statistically significant interaction was shown [Greenhouse-Geisser *F*_(1.5, 77.9)_ = 0.971, *p* = 0.361, partial η^2^ = 0.018].

In the distinction between the AIS located directly below the optodes and those outside the direct measurement area, the 2 × 3 mixed ANOVA for *group*_*HealthyHem*_ (healthy hemisphere stroke, control group) × *upper body position* (A 30°, B 0°, and C 30°) revealed no statistically significant interaction between the two measurement locations over the different positions [*F*_(4, 102)_ = 1.409, *p* = 0.236, partial η^2^ = 0.052].

**Figure 4:**
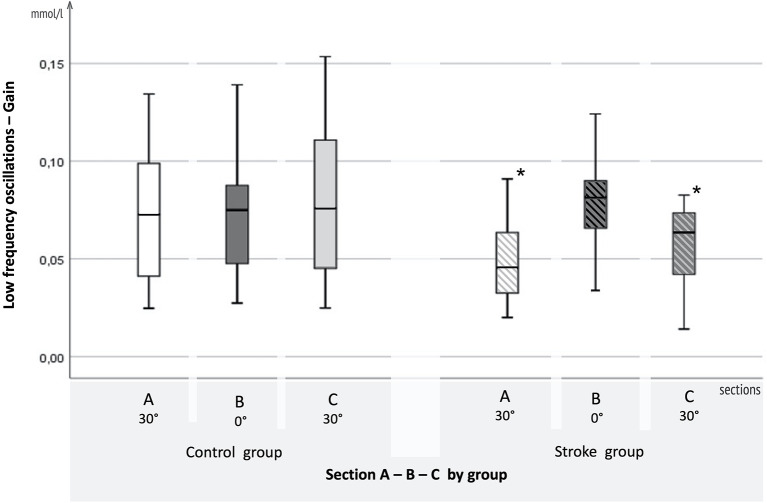
Results for LFO. Boxplots of adjusted gain in the low-frequency oscillation range for the AIS and control groups. On the *x*-axis, 3-, 5-min sections A (30°), B (0°), and C (30°), are displayed for the control group on the left and the AIS group on the right. Gain in mmol/l is plotted on the y-axis.

Descriptively (see Figure 4), gain does not differ for the control group over the course of the different upper body positions. However, in the stroke group, gain is increased for upper body position of 30° (sections A and C) compared to the supine position (0°, section B).

Statistically, regarding the LFO gain, the mixed ANOVA with between-subjects factor *group*_*AISHem*_ (stroke, controls) and within-subjects factor *upper body position* (A 30°, B 0°, and C 30°) showed a significant interaction effect [*F*_(1.903, 89.453)_ = 4.540, *p* = 0.015, partial η^2^ = 0.088]. There was a significant main effect for the groups differing in sections A (30°) (*p* = 0.026) and C (30°) (*p* = 0.013). The effect of the upper body position was significant in the stroke group [Greenhouse-Geisser *F*_(1.534, 36.6811)_ = 4.001, *p* < 0.037, partial η^2^ = 0.143]. In the healthy control group, changes in upper body position had no significant effect [Greenhouse-Geisser *F*_(1.820, 41.859)_ = 0.780, *p* > 0.05, partial η^2^ = 0.033]. For phase shift, the mixed ANOVA revealed no significant effect [Greenhouse-Geisser *F*_(2, 104)_ = 1.928, *p* = 0.141, partial η^2^ = 0.036].

In the upper VLFO range, the 2 × 3 mixed ANOVA for *group*_*AISHem*_ (stroke, controls) × *upper body position* (A 30°/B 0°/C 30°) showed no significant interaction in upper VLFO range for gain [Greenhouse Geisser *F*_(2, 104)_ = 0.520, *p* = 0.596, partial η^2^ = 0.010] or phase shift [*F*_(1.972, 92.670)_ = 0.233, *p* ≥ 0.05, partial η^2^ = 0.01).

As for the upper VLFO, the 2 × 3 mixed ANOVA for *group*_*AISHem*_ (stroke, controls) × *upper body position* (A 30°/B 0°/C 30°) confirms no significant interaction in lower VLFO for gain [Greenhouse Geisser *F*_(2, 104)_ = 4.361, *p* = 0.015, partial η^2^ = 0.077] or phase shift [Greenhouse Geisser *F*_(2, 104)_ = 1.358, *p* = 0.262, partial η^2^ = 0.025].

## Discussion

The present study showed that measuring CA in AIS patients in one single instrument with NIRS long- and short channels is feasible. Here, long channels were used to assess CA, while short channels were used to simultaneously observe systemic BP fluctuation. Furthermore, it was found that after comparing the intra- and extracranial oscillation signals with DFT, gain was significantly reduced in the stroke group compared to controls for (i) steady HOB elevation to 30°, and (ii) abrupt HOB elevation to 30°. Thus, in the stroke group CA did not modify systemic BP oscillations to the same extent as in the control group and impaired CA made cerebral oscillations passively follow the systemic BP oscillations (37).

In line with previous studies on acute stroke, our results confirm an impairment of CA in AIS (12, 26, 40, 57). However, reference studies so far only refer to measurements at rest without positioning maneuvers as a stressor.

Zhang et al. showed changes in cerebral blood flow in association with changes in arterial pressure after deflating a thigh cuff in 10 healthy subjects. The research group measured changes in velocity 10 s before and 20 s after the cuff deflation. Their results could either be caused by changes in arterial pressure or show a constant cerebral blood flow with active changes in the diameter of cerebral arteries and compensatory changes in resistance in downstream small vessels (58). The latter would be in line with our results in the AIS group. Nevertheless, to avoid a signal contamination during position changes, the first and last 30 s after position change were excluded from the analysis. Moreover, we included a healthy control group. The changes in LFO in our analyses are only displayed in the AIS group but not in the healthy control group.

The difference was seen in the LFO range. This frequency ranges around ~0.1 Hz and could be connected to the local myogenic activity in the terminal arterioles with influence of the sympathetic nervous system (28, 29). According to prevailing oscillation theories (17), there are two possible explanations for the reduced gain in stroke. It indicates either reduced autonomic nervous system (ANS) activity [pacemaker theory (29)] or reduced myogenic autoregulatory activity [baroreceptor reflex theory (59)]. However, a reduced ANS activity is not in line with the general findings on the ANS in acute stroke (7). Instead, it is assumed that the sympathetic nervous system, as part of the ANS, is overactive after stroke and dominates the parasympathetic nervous system (7). This makes the pacemaker theory the less plausible reason for gain-reduction.

On the other hand, the baroreceptor reflex theory with lowered myogenic autoregulatory activity seems plausible as a cause: after AIS the smooth muscles lose their tone, known as vasomotor paralysis (2). An intact capacity for vasoconstriction is necessary for the sympathetic nervous system to increase LFO gain (17). Baroreceptor reflexes have been confirmed to influence LFO in studies of animals subjected to surgical denervation of sinoaortic baroreceptors, with either severe attenuation or abolition of LFOs (60–62). Deactivation of the baroreceptor reflex with alpha-adreno blockers shows a similar pattern in humans (63, 64). Thus, reduced LFO-gain in AIS is most likely an indication of a disturbed local myogenic activity in the terminal arterioles, e.g., in the context of vasomotor paralysis.

This study shows that the impaired CA due to disturbed myogenic activity is detectable within the first 24 h after stroke. Results from Li et al. on CA in chronic stroke suggest that this paralysis may be permanent, as it was detected 12 months after stroke (65).

It was particularly the gain revealing significant results, but not the phase shift. Unfortunately, comparable studies on phase shift, which could be helpful for the interpretation of the discrepancy, have not been found. However, the reproducibility of LFO gain is remarkably high, especially in the short-term (66), and seems to be a much more robust parameter than phase shift for CA interpretation (67). Furthermore, the shorter oscillation length in the LFO range leads to more recorded oscillations within the measuring period. Thus, more data that can be obtained within the same time than in the VLFO range. Accordingly, a significant result would be displayed most clearly in the LFO range.

The gain reduction in stroke patients occurred especially with an upright upper body position (sections A and C). One reason could be that the effects of pathological vasomotor paralysis are particularly noticeable in the upright position. This could arise from orthostasis reaction, and the additional work required against the force of gravity, which further impairs the already sensitively disturbed CA.

We could not show any difference between measurements directly over the AIS area and those next to the AIS area in the stroke group. Therefore, gain reduction occurs not only in the impaired brain tissue but also in its penumbra. Still, this effect only occurred in the affected hemisphere: by analyzing the unaffected hemisphere of stroke patients compared to the control group, a selection bias of the stroke group could be ruled out as the cause of the gain-reduction. Likewise, in literature, measurements of CA in healthy subjects showed identical between their two hemispheres (68). In contrast, CA in stroke remained largely intact in the unaffected hemisphere, even in large-scale strokes [see meta-analysis by Intharakham et al. (69)]. In line with this, our analyses suggest that CA in acute stroke is primarily impaired in the affected hemisphere.

Moreover, the present study could demonstrate that AIS does not affect the metabolic activity in the cerebral capillary network as part of the CA (36) (cf. lower VLFO). Unfortunately, comparable NIRS studies in this frequency range were unavailable for acute stroke.

Furthermore, the given results demonstrate that AIS does not affect the neurogenic activity of the large arteries as one CA mechanism (31–33) (cf. upper VLFO). However, it should be noted that changes in CO2 partial pressure can affect upper VLFO (34, 35). To exclude this confounder, in the present study subjects underwent a detailed cardiopulmonary examination, and vital signs were taken prior to the measurement. In case of respiratory abnormalities, the measurements were postponed, or the patient was excluded. However, to the best of our knowledge, no studies (including the present one) performed blood gas analysis before recording, the method of choice for determining pCO2 (65).

Additionally, this study showed that CA in healthy subjects could adequately compensate for systemic changes in BP and perfusion during changes in position. This confirms in literature where healthy persons showed no differences in CA between left and right hemispheres, across gender, and over time (68) with resilience to visual (39), respiratory (70), motoric (71), or positional stimuli (28).

One limitation of NIRS is the modest depth penetration of near-infrared light. Therefore, NIRS does not capture deep subcortical activation and is limited to superficial brain regions (72). Another general limitation is the relatively low NIRS signal-to-noise ratio, mainly resulting from contamination by systemic noise resulting in the development of hardware-based solutions like short channels for dealing with physiological noise available (73, 74). However, these developments made studies like the present one possible in the first place. Moreover, a constant blood pressure measurement was not performed during the NIRS-recordings. Though, the patients' complete vital sign status was obtained at close intervals before and after the examination.

Further studies are suggested to deepen the hypothesis of impaired arterial myogenic regulation in AIS patients. For example, the commonly used hand-raising test in combination with a finger blood volume pulse (F-BVP) could be used to investigate whether arterial myogenic regulation differs between controls and AIS patients.

## Conclusion

The present study demonstrates impaired CA in AIS when the upper body is elevated 30° as well as after abrupt upper body elevation. Moreover, this study shows that CA in healthy subjects could adequately compensate for systemic changes in BP and perfusion during changes in position. Also, a new method of using NIRS short channels to measure cerebral autoregulation in one single instrument feasible to administrate in an AIS setting could be demonstrated. It could be shown that short channels can simultaneously record systemic BP signals next to the intracranial long channel. In combination with long channel data, short channels can effectively assess the functional capacity of CA in AIS. Characteristic stroke-related distortion in CA, their biological characteristics, and diagnostic relevance should be tested in future studies.

## Data availability statement

The raw data supporting the conclusions of this article will be made available by the authors, without undue reservation.

## Ethics statement

The studies involving human participants were reviewed and approved by Medical Ethics Committee, University of Oldenburg. The patients/participants provided their written informed consent to participate in this study.

## Author contributions

KW conceived and designed the research and made a critical revision of the manuscript for important intellectual content. SB conceived and designed the research, acquired the data, analyzed, interpreted the data, performed the statistical analysis, and drafted the manuscript. FK, KK, CM, and TL made a critical revision of the manuscript for important intellectual content. All authors have made substantial contributions to the manuscript. All authors contributed to the article and approved the submitted version.

## Conflict of interest

The authors declare that the research was conducted in the absence of any commercial or financial relationships that could be construed as a potential conflict of interest.

## Publisher's note

All claims expressed in this article are solely those of the authors and do not necessarily represent those of their affiliated organizations, or those of the publisher, the editors and the reviewers. Any product that may be evaluated in this article, or claim that may be made by its manufacturer, is not guaranteed or endorsed by the publisher.
