# Supplementary material for: Assessment of dynamic cerebral autoregulation in near-infrared spectroscopy using short channels: A feasibility study in acute ischemic stroke patients
Source: Front Neurol. 2022 Nov 21;13:1028864. doi: 10.3389/fneur.2022.1028864 (PMC9719939; doi:10.3389/fneur.2022.1028864)

Supplementary Material

# Boxplots Low Frequency Oscillations

**Figure 1.** Boxplots of phase shift in the low frequency oscillation range for the AIS and control groups. On the x-axis, three five-minute sections A (30°), B (0°), and C (30°), are displayed for the control group on the left and the AIS group on the right.


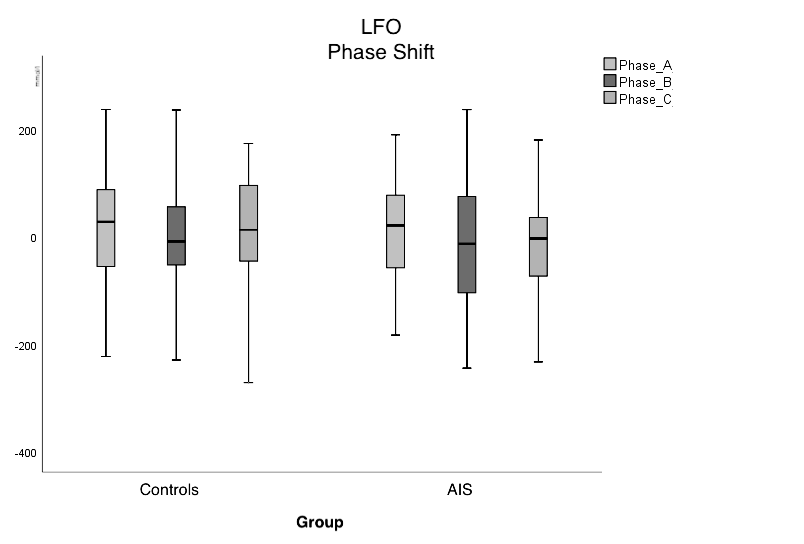


# Boxplots Upper Very Low Frequency Oscillations

**Figure 2.** Boxplots of adj. gain in the upper very low frequency oscillation range for the AIS and control groups. On the x-axis, three five-minute sections A (30°), B (0°), and C (30°), are displayed for the control group on the left and the AIS group on the right.


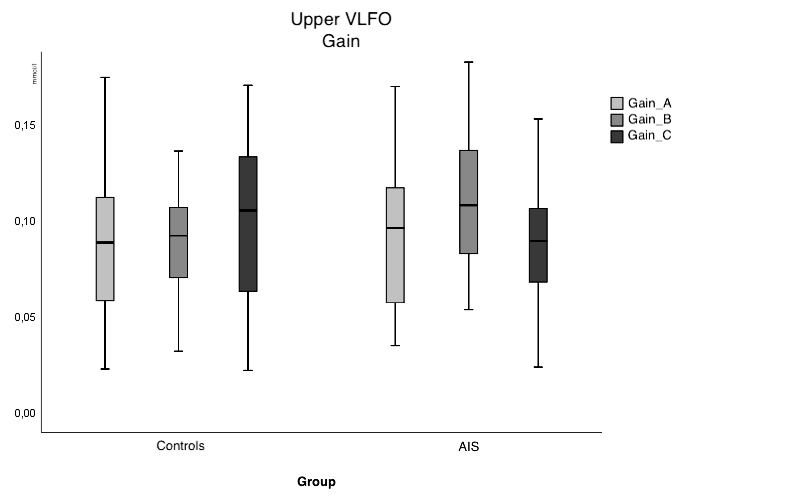


**Figure 3.** Boxplots of phase shift in the upper very low frequency oscillation range for the AIS and control groups. On the x-axis, three five-minute sections A (30°), B (0°), and C (30°), are displayed for the control group on the left and the AIS group on the right.
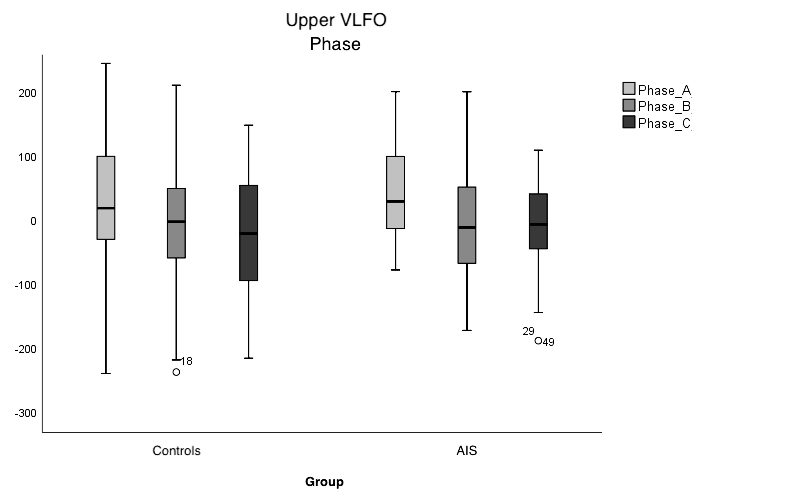


# Boxplots Lower Very Low Frequency Oscillations

**Figure 4.** Boxplots of adj. gain in the lower very low frequency oscillation range for the AIS and control groups. On the x-axis, three five-minute sections A (30°), B (0°), and C (30°), are displayed for the control group on the left and the AIS group on the right.
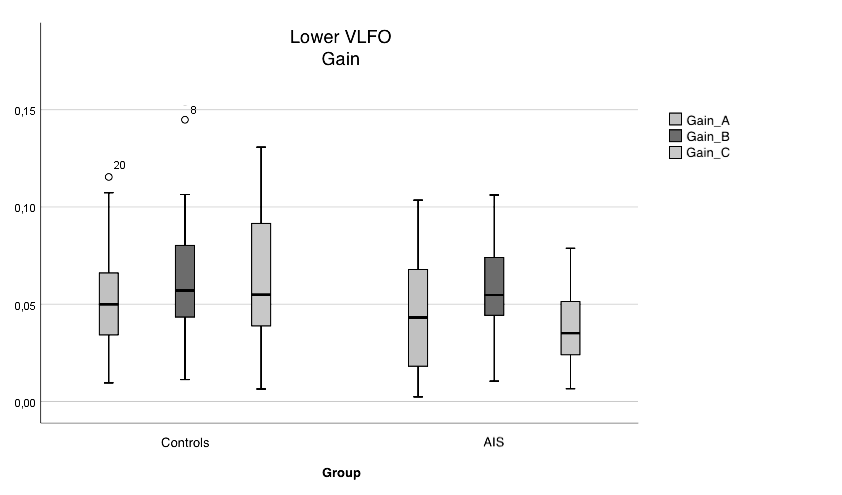


**Figure 5.** Boxplots of phase shift in the lower very low frequency oscillation range for the AIS and control groups. On the x-axis, three five-minute sections A (30°), B (0°), and C (30°), are displayed for the control group on the left and the AIS group on the right.
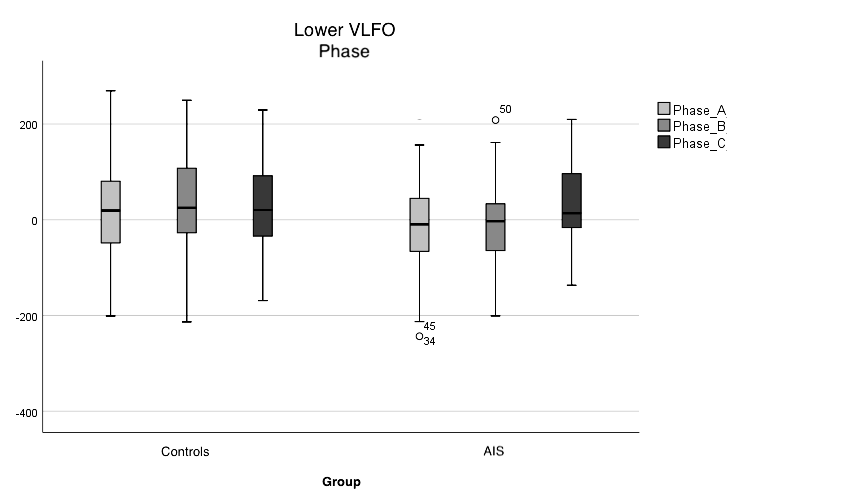

Supplement: Supplementary file 1 [file Table_1.DOCX]
